# Effects of *Lactobacillus reuteri* supplementation on the gut microbiota in extremely preterm infants in a randomized placebo-controlled trial

**DOI:** 10.1016/j.xcrm.2021.100206

**Published:** 2021-02-22

**Authors:** Magalí Martí, Johanne E. Spreckels, Purnika Damindi Ranasinghe, Erik Wejryd, Giovanna Marchini, Eva Sverremark-Ekström, Maria C. Jenmalm, Thomas Abrahamsson

**Affiliations:** 1 Department of Biomedical and Clinical Sciences, Linköping University, Linköping, Sweden; 2 Department of Genetics, University Medical Centre Groningen, Groningen, the Netherlands; 3 Institute for Global Food Security, School of Biological Sciences, Queen’s University Belfast, Belfast, UK; 4 Department of Paediatrics, Linköping University, Linköping, Sweden; 5 Department of Neonatology, Astrid Lindgren Children’s Hospital, Karolinska University Hospital and Institute, Stockholm, Sweden; 6 Department of Molecular Biosciences, The Wenner-Gren Institute, Stockholm University, Stockholm, Sweden

**Keywords:** microbiota, microbial diversity, preterm infant, extremely low birth weight infant, probiotic, supplementation, *Lactobacillus*, *Staphylococcus*, randomized controlled trial, necrotizing enterocolitis

## Abstract

Extremely low birth weight (ELBW) infants often develop an altered gut microbiota composition, which is related to clinical complications, such as necrotizing enterocolitis and sepsis. Probiotic supplementation may reduce these complications, and modulation of the gut microbiome is a potential mechanism underlying the probiotic effectiveness. In a randomized, double-blind, placebo-controlled trial, we assessed the effect of *Lactobacillus reuteri* supplementation, from birth to post-menstrual week (PMW)36, on infant gut microbiota. We performed 16S amplicon sequencing in 558 stool samples from 132 ELBW preterm infants at 1 week, 2 weeks, 3 weeks, 4 weeks, PMW36, and 2 years. Probiotic supplementation results in increased bacterial diversity and increased *L. reuteri* abundance during the 1^st^ month. At 1 week, probiotic supplementation also results in a lower abundance of Enterobacteriaceae and Staphylococcaceae. No effects were found at 2 years. In conclusion, probiotics may exert benefits by modulating the gut microbiota composition during the 1^st^ month in ELBW infants.

## Highlights

*L. reuteri DSM 17938* dominates the microbiota in supplemented ELBW infants
Supplemented ELBW infants have higher gut microbiota richness and diversity
Supplemented infants have lower abundance of *Enterobacteriaceae* and *Staphylococcaceae*
No effects of probiotic supplementation on the gut microbiota at PMW36 and 2 years

## Graphical abstract

**Figure:**
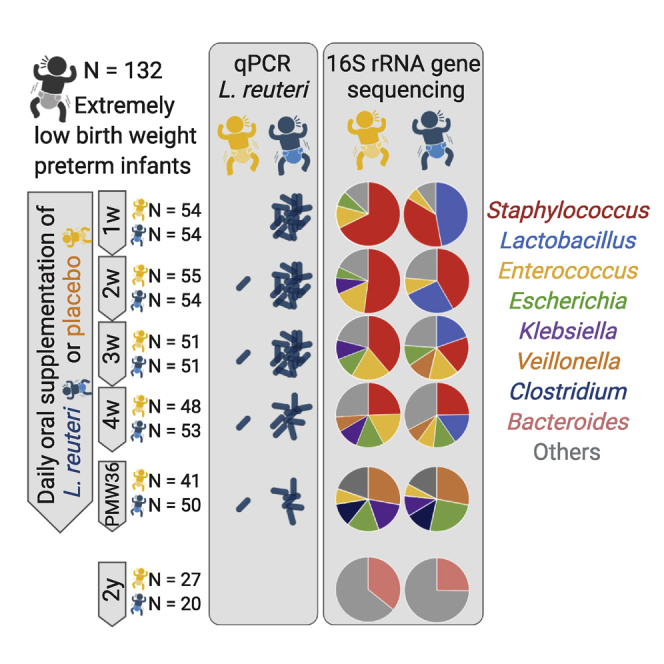

## Teaser

Martí et al. demonstrate, in a randomized, double-blind, placebo-controlled trial, that daily supplementation of *L. reuteri* from birth to post-menstrual week 36 in ELBW preterm infants modulates the gut microbiota composition, resulting in increased bacterial diversity and high abundance of the supplemented strain during the 1^st^ month of life.

## Introduction

Although the care of preterm infants has developed dramatically during the last decades, about 23% of extremely low birth weight (ELBW) (birth weight <1,000 g) preterm infants, born in Sweden, die due to clinical complications,^1^ such as necrotizing enterocolitis (NEC)^2^ and sepsis,^3^ and the survivors have a high risk of long-term neurological disabilities.^4^ The pathogenesis of such clinical complications is multifactorial and has been attributed to the immature development of the immune system, altered intestinal epithelial barrier function, gut motility, and regulation of the microvascular circulation as well as formula feeding and antibiotic treatment.^5^^,^^6^ Current research interest has also focused on the composition and function of the developing gut microbiota, because an altered bacterial composition and lower diversity may be a major risk factor linked to these severe clinical complications.^2^, ^3^, ^4^^,^^7^ For example, increased relative abundance of Proteobacteria (class Gammaproteobacteria or family Enterobacteriaceae), decreased relative abundance of Firmicutes and Bacteroidetes, absence of *Propionibacterium*, and an overall decrease in microbial richness have been reported to precede the onset of NEC.^2^^,^^8^

Probiotic supplementation to very low birth weight (VLBW) infants has been shown to reduce the risk of complications, such as NEC^9^ and late-onset of sepsis.^10^ The effect, however, seems to be strain dependent, and there is still insufficient evidence for an effect in ELBW infants.^9^^,^^10^ The strain *Lactobacillus reuteri* DSM 17938 has been reported to reduce sepsis, feeding intolerance, and days on antibiotic treatment and improve growth and immune function in randomized-controlled trials in preterm infants.^11^, ^12^, ^13^, ^14^ Recently, it was also reported to reduce NEC in preterm infants in a strain-specific systematic review.^15^

The underlying mechanisms of the effectiveness of probiotics are hypothesized to be modulation of the gut microbiome, reinforcement of the intestinal barrier, and interaction with the innate and adaptive immune system.^9^^,^^16^^,^^17^ Competitive exclusion of pathogens either via secretion of antimicrobial intermediaries or via inhibition of pathogenic adhesion is another mechanism generally associated to *L. reuteri*.^18^^,^^19^ Despite that an effect on the gut microbiota has been one of the main hypotheses on how probiotics act in preterm infants, there are still very few reports on the gut microbiome composition in VLBW infants during the 1^st^ month of life in randomized-controlled trials.

This study was part of a prospective randomized, double-blind, placebo-controlled, multi-center trial, in which 134 ELBW preterm infants were supplemented with either *L. reuteri* DSM 17938 or placebo daily from birth to post-menstrual week (PMW)36, evaluating feeding tolerance and growth.^13^ The primary aim of the present study was to investigate the effect of supplementation with *L. reuteri* DSM 17938 on the gut microbiota composition of ELBW preterm infants during the neonatal period and at a follow-up at 2 years of age. Secondary aims were to analyze the microbial composition in relation to NEC, sepsis, and growth rate. We hypothesized that *L. reuteri* supplementation would modulate the gut microbiota composition by, for example, enhancing the gut microbiota diversity and reducing opportunistic pathogens during the neonatal period.

## Results

### ELBW infant cohort

**Figure 1:**
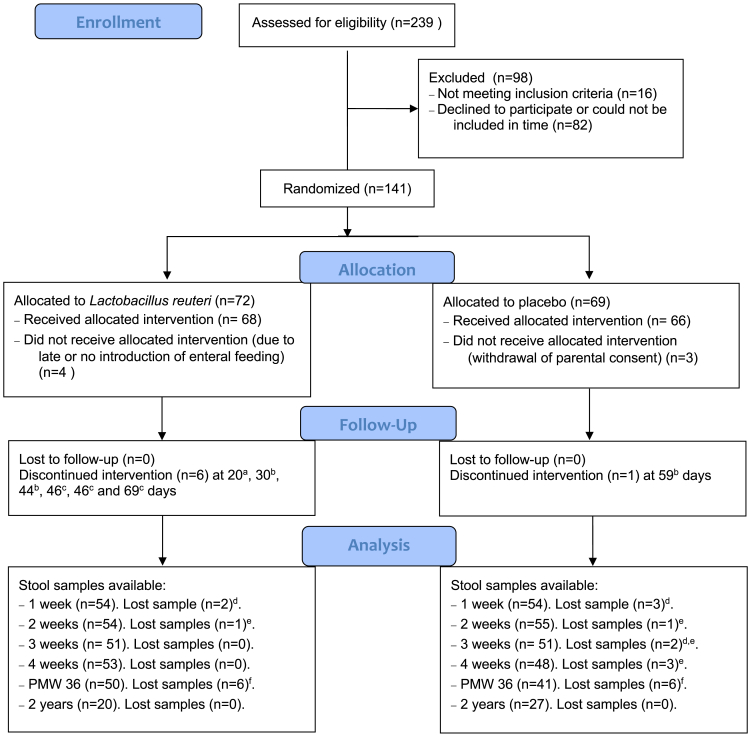
Flow chart of the study PMW36: post-menstrual week 36. ^a^Study product was discontinued by mistake after transfer to other hospital (n = 1). ^b^Study product was not administrated again by mistake after temporarily being withheld during nil oral (n = 3). ^c^Study product ran out temporarily at the study site (n = 3). ^d^Insufficient amounts of DNA were recovered from extraction (n = 6). ^e^Library preparation failed (n = 6). ^f^Sequencing failed (n = 12).

Overall, 132 infants were included in the study and a total of 558 stool samples were analyzed (Figure 1). The background and clinical characteristics of the infants included in the analyses at 1 week are displayed in Table 1 and at 2 weeks, 3 weeks, 4 weeks, PMW36, and 2 years in Table S1. The following clinical characteristics significantly differed between the *L. reuteri* and placebo groups: gender at 1 week and 3 weeks; delivery mode at 3 weeks; and treatment with antibiotics at 4 weeks, as well as chorioamnionitis, infants from multiple pregnancies, and total days on antibiotics at PMW36 (Table S1).

**Table 1:** Background and clinical characteristics of extremely low birth weight preterm infants from which stool samples were collected at 1 week of age

Variables	Statistical test	Placebo n = 54	*Lactobacillus reuteri* n = 54	p value
Gestational age, weeks, mean (SD)	Student’s t test	25.5 (1.3)	25.5 (1.3)	0.89
Birth weight, g, median (IQR)	Mann-Whitney U test	763 (197.8)	727.5 (172.2)	0.15
Birth weight *Z* score, median (IQR)	Mann-Whitney U test	−0.8 (1.3)	−0.9 (1.7)	0.25
Birth length, cm, median (IQR)	Mann-Whitney U test	33.5 (3.5)	33 (3.8)	0.22
Birth length *Z* score, median (IQR)	Mann-Whitney U test	−0.8 (1.6)	−1.6 (2.3)	0.06
Birth head circumference, cm, median (IQR)	Mann-Whitney U test	23 (3)	23 (2)	0.12
Birth head circumference *Z* score, mean (SD)	Student’s t test	−0.7 (0.8)	−1 (0.8)	0.06
Apgar score at 5 min, median (IQR)	Mann-Whitney U test	7 (2)	7 (4)	0.68
Apgar score at 10 min, median (IQR)	Mann-Whitney U test	8 (2)	8 (2.8)	0.34
Small for gestational age (weight <2 SD), n (%)	Pearson’s Χ^2^ test	10 (19%)	17 (31%)	0.18
Gender female/male, n (%)	Pearson’s Χ^2^ test	19 (35%)/35 (65%)	31 (57%)/23 (43%)	0.03
Infants from multiple pregnancy, n (%)	Pearson’s Χ^2^ test	17 (31%)	17 (31%)	1.00
Caesarean section, n (%)	Pearson’s Χ^2^ test	30 (56%)	40 (74%)	0.07
Maternal smoking, n (%)	Fisher’s exact test	4 (7%)	4 (7%)	1.00
Pre-eclampsia, n (%)	Fisher’s exact test	4 (7%)	5 (9%)	1.00
Chorioamnionitis, n (%)	Pearson’s Χ^2^ test	8 (15%)	14 (26%)	0.23
Preterm premature rupture of membranes, n (%)	Pearson’s Χ^2^ test	13 (24%)	19 (35%)	0.29
Maternal antibiotics, n (%)	Pearson’s Χ^2^ test	27 (50%)	33 (61%)	0.33
Antenatal corticosteroids, n (%)	Fisher’s exact test	53 (98%)	53 (98%)	1.00
Inclusion site—Stockholm/Linköping, n (%)	Pearson’s Χ^2^ test	35 (65%)/19 (35%)	35 (65%)/19 (35%)	1.00
Treatment with antibiotics within the actual week, n (%)	Pearson’s Χ^2^ test	54 (100%)	54 (100%)	NA
Total days on antibiotics, median (IQR)	Mann-Whitney U test	7 (0)	7 (0)	0.93
Total days with insulin, median (IQR)	Mann-Whitney U test	0 (0)	0 (0)	0.52
Insulin within the actual week, n (%)	Fisher’s exact test	9 (17%)	12 (22%)	0.63
Total days with corticosteroids, median (IQR)	Mann-Whitney U test	0 (0)	0 (0)	0.41
Corticosteroids within the actual week, n (%)	Fisher’s exact test	4 (7%)	2 (4%)	0.68

Apgar score is missing from one infant in the *L. reuteri* group. See Table S1 for all the 6 time points (1 week to 2 years of age). IQR, inter-quartile range; NA, not applicable.

### Microbial community composition and structure differs between the placebo and probiotic groups during the 1^st^ month of life

**Figure 2:**
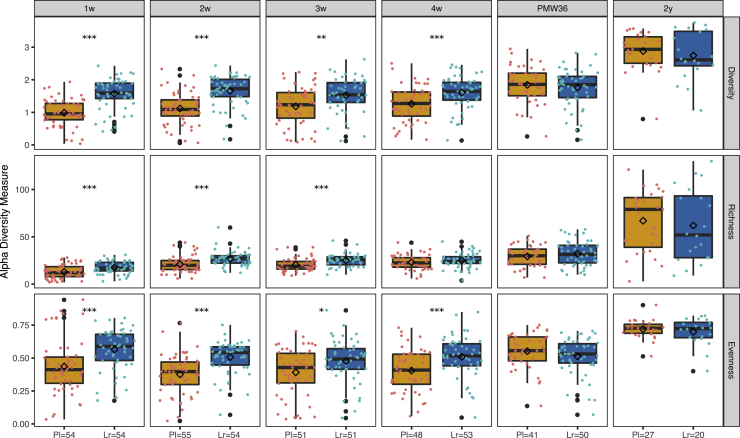
Gut microbiota α-diversity of ELBW preterm infants supplemented with *L. reuteri* or placebo Boxplots (median with 25% and 75% percentiles and 1.5× the interquartile range; diamond shape depicts the mean) showing the α-diversity (Shannon index), richness (observed ASVs), and evenness (Pielou’s evenness index) from 1 week to 2 years of life in ELBW preterm infants supplemented with *L. reuteri* (Lr) or placebo (Pl). PMW36, post-menstrual week 36; w, week; y, year. ∗∗∗p < 0.001, ∗∗p < 0.01, and ∗p < 0.05 with Mann-Whitney U test and p value adjustment for multiple comparisons with the method from Benjamini and Hochberg.

**Figure 3:**
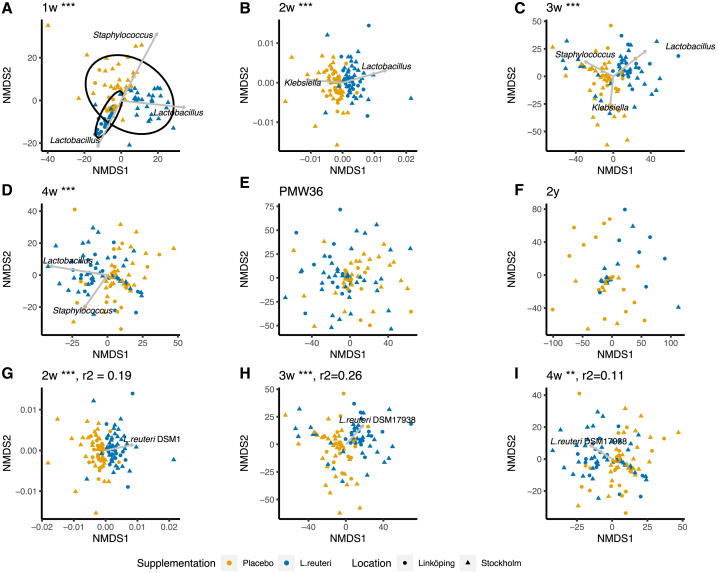
Clustering of the gut microbiota composition (β-diversity) of ELBW preterm infants supplemented with *L. reuteri* or placebo Non-metric multidimensional scaling (NMDS) of bacterial community composition from 1 week to 2 years of life across ELBW preterm infants supplemented with *L. reuteri* or placebo. (A–F) The ASVs that significantly contributed to the variance explained (*envfit();* p < 0.01 and R^2^ > 0.3) were classified at genus level, and only one genus for all ASVs pointing toward the same direction was displayed (Table S6). At 1 week, (A) the ellipses (confidence level 0.95) show Linköping and Stockholm because inclusion site also affected the bacterial community composition and *Lactobacillus* had different effects depending on the site. (G–I) The abundance of *L. reuteri* DSM 17938 (qPCR data) significantly (*envfit();* p < 0.01 and R^2^ > 0.3) correlated to bacterial community composition in the placebo group. Weight, length, and head circumference were adjusted for gestational age using the standard deviation score (*Z* score). ∗∗∗p < 0.001 with ANOSIM and p value adjustment for multiple comparisons with the method from Benjamini and Hochberg.

The *L. reuteri* group had significantly higher bacterial richness, diversity, and evenness than the placebo group during the 1^st^ month of life, except for richness at 4 weeks (Figure 2). The bacterial community composition (β-diversity) significantly differed between the two groups during the 1^st^ month of life (analysis of similarities [ANOSIM], p = 0.001; Figures 3A–3D). *Lactobacillus, Staphylococcus*, and *Klebsiella* were the main genera contributing to the differences in bacterial composition. *Lactobacillus* was clearly associated with the *L. reuteri* group during the 1^st^ month of life, although *Staphylococcus* and *Klebsiella* were associated with the placebo group at 1 week, 3 weeks, and 4 weeks and 2 weeks and 3 weeks, respectively. At 1 week, the inclusion site (Stockholm or Linköping) also affected the bacterial community composition (ANOSIM; p = 0.001). The difference between the two study groups was still significant after stratifying the data according to the inclusion site (ANOSIM; p = 0.001). At PMW36 and 2 years, there were no significant differences in bacterial community structure (α-diversity; Figure 2) and composition (β-diversity; Figures 3E and 3F) between the two study groups.

Among the potential confounding factors mentioned above, treatment with antibiotics at 4 weeks, but not the other factors, was significantly associated with lower richness and diversity (Table S2) as well as different bacterial community composition (β-diversity). After stratifying the data, diversity and evenness only differed significantly between the *L. reuteri* and placebo group in infants that received antibiotics at 4 weeks (Table S2).

### Lower relative abundance of Staphylococcaceae and Enterobacteriaceae in the probiotic group during the 1^st^ week of life

**Figure 4:**
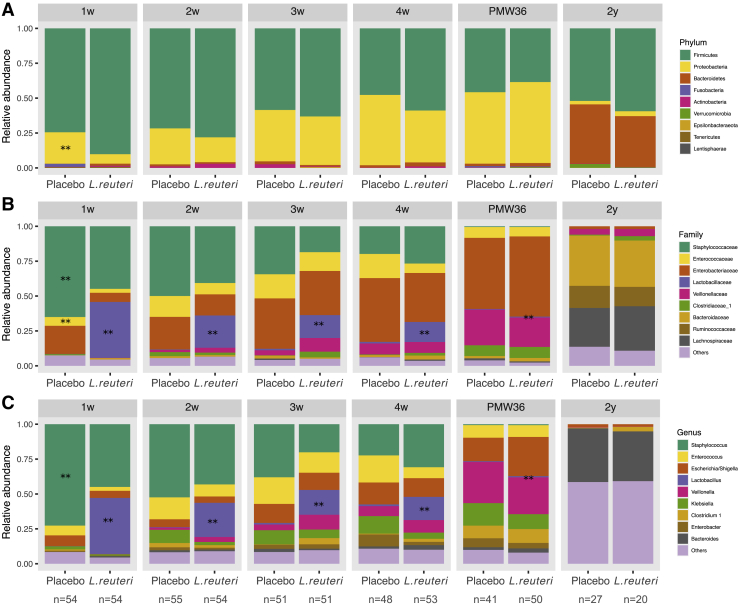
Taxonomic composition of the gut bacteria in ELBW preterm infants supplemented with *L. reuteri* or placebo Relative abundance of the dominant taxa is displayed at phylum (A), family (B), and genus (C) level. At family and genus levels, the taxa with a relative abundance of <1% across all samples and time points are grouped in “others.” ∗∗ indicates taxa that significantly differed in relative abundance between the *L. reuteri* and placebo groups (LEfSE; p = 0.01; Table S3).

We identified a total of 491, 566, 753, 798, 778, and 1,326 bacterial amplicon sequencing variants (ASVs) at 1 week, 2 weeks, 3 weeks, 4 weeks, PMW36, and 2 years, respectively. Firmicutes and Proteobacteria were the most abundant bacterial phyla during the neonatal period (1 week to PMW36), although Bacteroidetes increased and Proteobacteria decreased in relative abundance at 2 years (Figure 4A). *Staphylococcus* was the dominant genus in both study groups, and *Lactobacillus* was the second most abundant genus in the *L. reuteri* group during the 1^st^ month of life (Figure 4C), with both genera having a tendency toward decreasing in relative abundance over time. *Enterococcus* and *Escherichia/Shigella* also had a high relative abundance during the entire neonatal period, although *Veillonella* became dominant at PMW36. *Bacteroides* were present at very low levels during the neonatal period but dominated the bacterial community at 2 years in both study groups. *Bifidobacteria* were detected at low relative abundance (<1%) and thus belonged to the group “others” (Figure 4C).

Inference of differential relative abundance between the two study groups was analyzed with linear discriminant analysis effect size (LEfSE). At 1 week, the placebo group had significantly higher relative abundance of Proteobacteria at phylum level, Staphylococcaceae and Enterobacteriaceae at family level, and *Staphylococcus* at genus level, although the *L. reuteri* group had significantly higher abundance of *Lactobacillus* during the 1^st^ month of life and at PMW36 (Figure 4; Table S3). Among potential pathogens, only *Staphylococcus* significantly differed between the two groups. The prevalence and relative abundance of the most common pathogens found in the dataset are summarized in Table S4.

Inference of differential relative abundance between the two inclusion sites revealed that, at 1 week, the genera *Escherichia/Shigella* were significantly more abundant in Stockholm and, at 3 weeks, *Enterococcus* was significantly more abundant in Linköping. At 1 week, *Escherichia/Shigella* was detected in three infants (prevalence 8%) with a mean relative abundance of 1% in Linköping and in 29 infants (prevalence 41%) with a mean relative abundance of 7% in Stockholm. At 3 weeks, the prevalence of *Enterococcus* in Linköping was 95% (36 infants), although in Stockholm, it was 74% (52 infants), with a mean relative abundance of 27% and 9%, respectively.

### High abundance of *L. reuteri* DSM 17938 in the probiotic group

**Figure 5:**
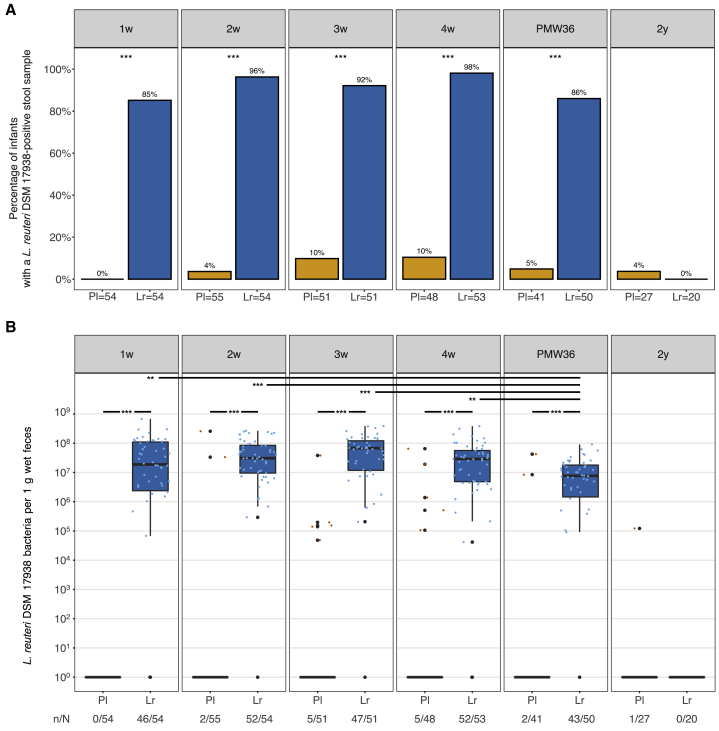
Prevalence and abundance of *L. reuteri* DSM 17938 Prevalence (A) and abundance (B) of supplemented *L. reuteri* DSM 17938 in *L. reuteri* and placebo groups at different time points. (A) Percentage of infants with a stool sample positive for the supplemented strain. (B) Boxplots (median with 25% and 75% percentiles and 1.5× the interquartile range) show the abundance as the number of *L. reuteri* DSM 17938 bacteria per 1 g wet feces. Colored dots indicate the *L. reuteri* DSM 17938 abundance in individual stool samples positive for the supplemented *L. reuteri* strain; the number of *L. reuteri* DSM 17938 bacteria per 1 g wet feces for infants with a *L. reuteri* negative stool sample was set to 1 for graphical display; (n) indicates the number of infants with a stool sample positive for supplemented *L. reuteri* DSM 17938; (N) indicates the total number of infants with a stool sample in the *L. reuteri* or placebo group at the indicated time point. Prevalence and abundance between groups were compared using Fisher’s exact tests and Mann-Whitney U tests, respectively, and adjusted for multiple comparisons with the method from Benjamini and Hochberg. Significant differences in *L. reuteri* DSM 17938 abundance in the *L. reuteri* group across neonatal time points (1 week to PMW36) were tested for with a Kruskal-Wallis test with Dunn post hoc test and p value adjustment for multiple comparisons with the method from Benjamini and Hochberg. ∗∗∗p < 0.001; ∗∗p < 0.01.

To specifically investigate the prevalence and abundance of the supplemented *L. reuteri* DSM 17938 in stool, we used strain-specific quantitative PCR (qPCR). During the neonatal period (1 week to PMW36), the prevalence of *L. reuteri* DSM 17938 was significantly higher in the *L. reuteri* compared to the placebo group (Figure 5A; Fisher’s exact test; adjusted p < 0.001). At 2 years, the strain was only detected in one infant from the placebo group. The abundance of *L. reuteri* DSM 17938 was also significantly higher in the *L. reuteri* group compared to the placebo group during the neonatal period (Figure 5B), and the abundance within the *L. reuteri* group was lower at PMW36 compared to 1 week, 2 weeks, 3 weeks, and 4 weeks (Figure 5B). Furthermore, *L. reuteri* DSM 17938 abundance was significantly associated with the gut bacterial community at 2 weeks, 3 weeks, and 4 weeks in the infants receiving the probiotic (Figures 3G–3I).

### Microbial biodiversity in relation to NEC, sepsis, and growth

Sub-analyses comparing the microbiota of NEC and sepsis cases with those of healthy controls were performed, because probiotics have been shown to reduce NEC and sepsis, and an altered bacterial composition and lower diversity have been reported to precede the onset of these diseases in preterm infants. In total, 15 out of 132 infants had NEC (eight in the placebo group and seven in the *L. reuteri* group), and 48 had culture-proven sepsis (23 in the placebo group and 25 in the *L. reuteri* group). Nine of the infants had both. Stool samples within 1 week prior to the onset of NEC were obtained from seven infants (four in the placebo group and three in the *L. reuteri* group) and prior to the onset of sepsis from 28 infants (14 in the placebo group and 14 in the *L. reuteri* group). The α-diversity and differential abundance of taxa in the NEC and sepsis cases were compared with two matched controls per index case. The selection criteria for the controls were, in a preferred order: supplementation; location; gender; gestational age; delivery mode; and treatment with antibiotics. There were no significant differences in α-diversity indices (Table S2) or relative abundance (Figure S1), with one exception: the genus *Clostridium* was significantly more relatively abundant in the sepsis group (LEfSE; p < 0.01).

**Figure 6:**
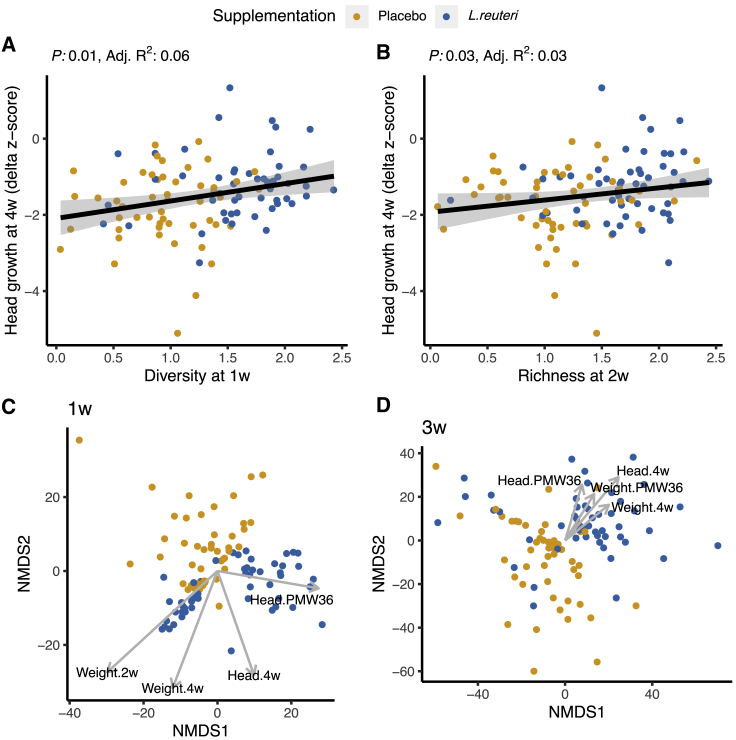
Correlation between growth parameters and ELBW preterm infant gut microbiota composition (A and B) Head growth until 4 weeks of life correlated to microbial diversity at 1 week (simple linear regression; p = 0.007; adjusted R^2^ = 0.06; A) and microbial richness at 2 weeks (simple linear regression; p = 0.035; adjusted R^2^ = 0.03; B). (C and D) Head (head) and weight (weight) growth rate significantly (*envfit()*; p < 0.05 and R^2^ 0.1–0.2) correlated to the microbial community composition at 1 week (C) and 3 weeks (D) of life.

In the original trial, the probiotic group had better head growth from birth to 4 weeks than the placebo group.^13^ In the present study, higher bacterial diversity at 1 week and higher richness at 2 weeks were associated with significantly better head growth rate at 4 weeks (Figures 6A and 6B) but explained only 6% and 3% of the variance, respectively. The bacterial composition at 1 week and 3 weeks was related to head growth at 4 weeks and PMW36 (R^2^ range = 7%–14%), as well as to weight gain at 2 weeks, 4 weeks, and PMW36 (R^2^ range = 6%–19%; Figures 6C and 6D). There was no correlation between the growth rate and the relative abundance of *L. reuteri* during the 1^st^ month of life (data not shown). Additionally, we applied the sparse microbial causal mediation model (SparseMCMM) in order to test whether the microbial community mediated the effect of probiotic supplementation on the growth parameters, but we found no significant mediation effect (Table S5).

## Discussion

This randomized-controlled study shows that infants supplemented daily with *L. reuteri* DSM 17938 had increased bacterial diversity and different bacterial community composition as compared to the infants in the placebo group. These differences did not remain after the 1^st^ month of life, i.e., at PMW36 and at the follow-up at 2 years. The prevalence of the supplemented strain was high during the neonatal period, and its abundance correlated with the infant gut bacterial composition at 2 weeks to 4 weeks. Moreover, *Lactobacillus spp.* dominated the gut microbiota in the probiotic group during the 1^st^ month of life and also correlated with the bacterial composition of the probiotic group, thus likely explaining most of the observed differences. This high relative abundance of *Lactobacillus*, and more specifically the supplemented *L. reuteri* DSM 17938, during the 1^st^ month of life in the probiotic group was probably due to a low gut bacterial load in ELBW infants during the 1^st^ weeks of life.^20^^,^^21^ Moreover, the high relative abundance of *Lactobacillus* at 1 week might also explain why there were significant differences in the relative abundance of other bacterial taxa. The decrease in the abundance of *L. reuteri* DSM 17938 at PMW36 despite the ongoing probiotic supplementation was most likely explained by the maturation of the colonic microbiota and an increase in anaerobic bacteria,^20^ resulting in increased competition for the niche and nutrients.^22^ Facultative anaerobes like *Streptococci* and *Lactobacilli* are the dominating taxa in the upper part of the human intestine, although obligate anaerobes are dominating the microbiota in the colon.^23^ Therefore, it is possible that *L. reuteri* continued to be a dominating strain in the small intestine, acting on the epithelium, also in the end of the neonatal period, despite the low levels in the stool samples.

The study is consistent with previous reports of an aberrant gut microbiota in VLBW infants during the neonatal period,^21^ including high relative abundance of Proteobacteria, low relative abundance of Bacteroidetes at phylum level, high relative abundance of *Staphylococcus*, *E. coli*, *Klebsiella*, and *Enterococcus* at genus level, and almost no *Bifidobacteria*, despite that the infants were fed exclusively with breast milk until they reached a weight of at least 2,000 g. The low abundance of *Bifidobacteria* in the preterm infants may be due to its susceptibility to the antibiotics frequently administered to these children,^24^ although it also has been proposed that gut immaturity and low gestational age in itself may explain the low prevalence of *Bifidobacteria* in preterm infants.^20^ Increased relative abundance of Proteobacteria and decreased relative abundance of Firmicutes and Bacteroidetes and a lack of *Propionibacterium* have been reported to precede the onset of NEC.^2^^,^^8^ Interestingly, despite only being significant at 1 week, the relative abundance of Proteobacteria and its family Enterobacteriaceae were lower in the probiotic group. Moreover, the difference in bacterial composition (β-diversity) between the two study groups was partially explained by *Klebsiella*, a genus in the Enterobacteriaceae family, in the placebo group during the 1^st^ month of life.

Importantly, the gut microbiota was assessed every week during the 1^st^ month of life in the present study, as this is the critical time window when most cases of sepsis, NEC, and feeding intolerance have their onsets in VLBW infants.^25^ There are two previous randomized-controlled trials in VLBW infants that have reported analyses of the gut microbiota composition. In the ProPrems trial, supplementation with two *Bifidobacterium* strains and a *Streptococcus* strain was associated with lower abundance of *Enterococcus*, although the analysis did not discriminate when exactly *Enterococcus* was lower during the neonatal period.^26^ In the PiPS trial, the gut microbiota was only analyzed at PMW36 and, as in the present study, there were no differences in diversity or bacterial taxa between the two study groups at that age.^27^ Recently, an observational study in which infants born before gestational week 34 were supplemented with a combination of *Bifidobacterium* and *Lactobacillus* confirmed our result.^28^ Besides an increased relative abundance of the supplemented *Bifidobacterium* and *Lactobacillus* strains, they reported a reduction of genera within the Enterobacteriaceae family during the whole neonatal period and a weak reduction of *Clostridium* in the end of the neonatal period. Although being consistent with our results, the differences between the two study groups seemed to be larger than in our trial. However, the case mix (i.e., differences in selection criteria, background factors, and treatments) also differed from our trial by including infants born at a later gestational age, and more importantly, their control infants were recruited from other hospitals than the supplemented infants.

Our study showed that gut microbiota can differ between two inclusion sites, which emphasizes the importance of a prospective randomized-controlled design with a stratification by inclusion site as, for example, nutrition could differ somewhat between different clinics. Another potential confounder of the probiotic intervention was antibiotic treatment, which was very common during the first 4 weeks of life in this cohort. Rougé et al.^24^ suggested that antibiotic treatment hindered colonization of the probiotic strains *Bifidobacterium longum* BB536 and *Lactobacillus rhamnosus* GG in ELBW infants, leading to a lack of effect of the probiotic supplement on time to reach full enteral feeding. Also, Costeloe et al.^29^ described an association of antibiotic treatment with decreased colonization by probiotic *Bifidobacterium breve* BBG-001 and a lack of effect on morbidities in very preterm infants in the PiPS trial. In contrast, the abundance of *L. reuteri* DSM 17938 did not seem to be impeded by antibiotic treatment in this study, which may be explained by its intrinsic resistance against a variety of antibiotic agents, including aminoglycosides and vancomycin.^30^

Cross-contamination has been proposed to explain the lack of effect in probiotic trials, such as the PiPS study.^29^ Indeed, *L. reuteri* was detected in the placebo group in the present study but only rarely and at very low levels as compared to the probiotic group. Thus, it is unlikely that cross-contamination caused a false negative effect in this study.

The original trial did not find any effect of *L. reuteri* supplementation on the primary clinical outcome feeding tolerance, but probiotic-supplemented infants had a better growth rate of the head during the 1^st^ month of life compared to the placebo group.^13^ The gut microbiota has been proven to affect growth and brain function in gnotobiotic animal models^31^^,^^32^ and has been associated with obesity in adults^33^^,^^34^ and growth in infants.^35^ The growth rate during the 1^st^ month of life had a significant but weak correlation with the microbiota composition and structure but no correlation with *L. reuteri*. Taxonomic characterization by itself, however, is unable to reveal the functional potential of the microbiota in preterm infants, which is necessary to understand the mechanisms underlying the effects of the gut microbiome on growth and nutrition.^36^ Future metatranscriptomic and metabolic profiling of the microbiota in relation to growth in ELBW infants is therefore warranted.

In conclusion, daily supplementation of *L. reuteri* DSM 17938 in ELBW preterm infants modulated the gut bacterial composition, with increased bacterial diversity and a high abundance of the supplemented probiotic during the 1^st^ month of life. Major effects on the other bacterial taxa were only observed during the 1^st^ weeks of life, with lower relative abundance of Staphylococcaceae and Enterobacteriaceae in the probiotic than the placebo group. No differences in the gut microbiota composition remained at the follow-up at 2 years of age.

### Limitations of study

A limitation of our study is that only stool samples were collected and analyzed, although many of the interactions between the gut microbiota and the intestinal mucosa and immune system are taking place in the small intestine. However, it would have been unethical to obtain samples invasively from ELBW preterm infants. The supplemented *L. reuteri* strain has been identified in intestinal biopsy specimens from ileum in adults, where it was seen to interact with the immune system by inducing a significantly higher amount of CD4-positive T lymphocytes in the ileal epithelium,^37^ although *in vitro* studies have shown *L. reuteri* to prime dendritic cells to produce increased levels of anti-inflammatory interleukin-10 (IL-10) and inhibit the proliferation of bystander effector T cells.^38^ Another limitation is that neither the original trial nor this sub-study were designed and powered to detect any significant effect on NEC or sepsis, and thus, findings from previous trials^2^^,^^8^ could not be confirmed in the present study. Moreover, we intentionally only included ELBW infants in this study, and thus, it limits the generalization of our findings to this patient group. A methodological limitation is that 16S rRNA gene sequencing produces compositional data and limits the analysis to relative abundances of bacterial taxa. To at least partially overcome this limitation, we applied qPCR to quantify the absolute abundance of the supplemented probiotic. Another limitation of the 16S rRNA gene sequencing data is that the taxonomical classification is limited in its accuracy to assign species.

## STAR★Methods

### Key Resources Table

**Table:** 

REAGENT or RESOURCE	SOURCE	IDENTIFIER
**Bacterial and virus strains**		

*Lactobacillus reuteri* DSM 17938 for probiotic use	BioGaia AB	https://www.biogaia.com/product-country/product-country-2163/
*Lactobacillus reuteri* DSM 17938 for DNA extraction to generate the standards for qPCR runs	Stefan Roos, Swedish Department of Molecular Sciences, University of Agricultural Sciences	N/A
DNA mock control ATCC MSA-2002	http://www.atcc.org/?geo_country=us	20 Strain Even Mix Whole Cell Material (ATCC® MSA 2002TM)

**Biological samples**		

Preterm infant stool samples (Table S10: metadata)	This paper	N/A

**Critical commercial assays**		

QIAamp PowerFecal DNA kit (50 preps)	QIAGEN	Cat No./ID: 12830-50
16S Metagenomic Sequencing Library Preparation	Illumina	Part # 15044223 Rev. B
Nextera XT Index kit v 2 (96 indexes, 384 samples)	Illumina	FC-131-2001
Agencourt AMPure XP, 450 mL	Beckman Coulter	A63882
2xKAPA HiFi HotStart ReadyMix	Roche	KK2601
PhiX Control Kit v3	Illumina	FC-110-3001
MiSeq Reagent Kit v3 (600-cycle)	Illumina	MS-102-3003
EZ1 DNA Tissue Kit	QIAGEN	Cat# 953034
SsoFast™ EvaGreen® Supermix	Bio-Rad	Cat# 1725201

**Deposited data**		

Fastq.gz files from 16S rRNA gene sequencing	This paper	Accession number ENA: PRJEB3653
Metadata	This paper	Table S10

**Oligonucleotides**		

16S rRNA primers pair 341F/805R (See 16S Metagenomic Sequencing Library Preparation)	Klindworth et al.^39^	N/A
*L. reuteri* 1694 gene forward primer 5¢-TTAAGGATGCAAACCCGAAC-3¢	Romani et al.^40^	N/A
*L. reuteri* 1694 gene reverse primer 5¢- CCTTGTCACCTGGAACCACT −3¢	Romani et al.^40^	N/A

**Software and algorithms**		

CFX Manager™ Software version 3.1	Bio-Rad	Cat# 1845000
bbduk.sh bbmap/38.08	Bushnell^41^	https://sourceforge.net/projects/bbmap/
FastQC/0.11.5 and MultiQC/1.7	Ewels et al.^42^	https://github.com/ewels/MultiQC
DADA2 Pipeline Tuytorial Dada2 version1.10.1	Callahan et al.^43^	https://benjjneb.github.io/dada2/tutorial.html
SILVA database version 132	German Network for Bioinformatics Infrastructure	https://www.arb-silva.de/documentation/release-123/
R Console 3.5.0	The R project for Statistical Computing	https://cran.r-project.org/bin/macosx/
DESeq2 R package version 1.28.2	Love et al.^44^	https://bioconductor.org/packages/release/bioc/html/DESeq2.html
diverse R package version 0.1.5	Guevaraet al.^45^	https://github.com/mguevara/diverse
phyloseq R package version 1.32.0	McMurdie and Holmes^46^	https://github.com/joey711/phyloseq

**Other**		

Placebo (Maltodextrin in oil suspension)	BioGaia AB	N/A

### Resource availability

#### Lead contact

Further information and requests for resources and reagents should be directed to and will be fulfilled by the Lead Contact, Magalí Martí (magali.marti.genero@liu.se).

#### Materials availability

This study did not generate new unique reagents.

#### Data and code availability

The 16S rRNA dataset generated during this study is available at the European Nucleotide Archive : PRJEB36531. The code generated during this study is available at: https://github.com/magge30/PROPEL-ELBW-16S.

Infant metadata, qPCR data and ENA accession numbers are included in Table S10.

### Experimental model and subject details

#### Human preterm infant cohort

134 infants born between gestational age 23+0 and 27+6 with a birth weight below 1,000 g were enrolled between 2012 and 2015 at two level III neonatal intensive care units (NICUs) (Astrid Lindgren Children’s Hospital, Stockholm, and Linköping University Hospital, Linköping, Sweden). See Table S1 for the background and clinical characteristics of the infants included in the study.

#### Human study design

The present study was part of a prospective randomized, double-blind, placebo-controlled, multi-center trial evaluating the effect of oral supplementation with the probiotic strain *L. reuteri* DSM 17938 in ELBW infants. The clinical outcomes have been published before^13^. Randomization was stratified by study center. The infants were characterized using comprehensive clinical data in a study-specific case report form from birth until PMW36 (Table 1). Weight, length and head circumference were recorded at birth, two weeks of age (2w), four weeks of age (4w), and at PMW36. In order to adjust for gestational age, the standard deviation score (z-score) for each measurement was calculated using Niklasson’s growth chart^47^. Growth rate was calculated as the difference in z-score between the later measurements and birth. Necrotising enterocolitis was staged according to Bell’s criteria^48^, and all cases of stage II or greater were recorded. A sepsis diagnosis required positive blood and/or cerebral spinal fluid culture, clinical deterioration and laboratory inflammatory response. Due to the 100% coverage of breast milk donor banks all infants were fed exclusively with breast milk until they reached a weight of at least 2,000 g. Protein and lipid fortification was based individually on analyses of the macronutrient and energy content of the breast milk given to each infant. Oral feeding started during the first day of life and increased gradually at a rate specified in clinical guidelines. Mother’s own milk and/or donor milk was analyzed for macronutrient and energy content. Targeted fortification was based on the guidelines of the European Society of Paediatric Gastroenterology, Hepatology and Nutrition (ESPGHAN)^49^. Breast milk fortification with bovine protein fortifier started when the enteral feeds had reached 100 mL/kg/day. The conduct complied with the principles of the International Conference on Harmonisation guidelines for Good Clinical Practice (ICH-GCP).

A total of 558 stool samples, collected from 132 infants, were included in the study. Samples were collected weekly during the first four weeks of life (1w, 2w, 3w and 4w), at PMW36, and at a follow-up at 2y (Figure 1). The samples were stored in sterile tubes at −20°C (short-term) and subsequently at −80°C until analysis. The scientist performing the microbial analyses was blinded until statistical analyses started.

#### Supplementation

Daily supplementation of *L. reuteri* DSM 17938 (1.25 × 10^8^ bacteria/day) or placebo started within three days of age and continued until PMW36. *L. reuteri* DSM 17938 was provided in oil drops consisting of sunflower oil, medium chain triglyceride oil and silicon dioxide. The placebo was maltodextrin provided in an identical oil suspension and it was not possible to differentiate the placebo from the active product by smell, taste or visual appearance. The study product was administered via the gastric tube or via mouth (if the nasogastric tube had been removed) but was withheld during periods when infants were nil orally. The drops were flushed down by at least 0.3 mL breast milk after the administration in the gastric tube. The study products were provided by BioGaia AB (Stockholm, Sweden) in identical oil suspensions. The strain *L. reuteri* DSM 17938 has been derived from the strain *L. reuteri* ATCC 55730 by removing two plasmids carrying *tet*(W) tetracyclin and *lnu*(A) lincosamide resistance genes^50^. Originally the mother strain *L. reuteri* ATCC 55730 was isolated from the breast milk of a Peruvian mother. The manufacturer checked the quality of the study product regularly, and the concentration of *L. reuteri* was within the stipulated limits in all batches used in the trial.

#### Ethical approval

Written informed consent was obtained from both parents. The study was approved by the Ethics Committee for Human Research in Linköping, Sweden (Dnr 2012/28-31, Dnr 2012/433-32).

### Method details

#### DNA extraction of infant stool samples

Total DNA was extracted from 0.10 ± 0.03 g of stool samples using the QIAamp PowerFecal DNA kit (QIAGEN, Hilden, Germany) on the QIAcube instrument (QIAGEN) according to the manufacturer’s instructions, with slight modifications: after heating, samples were disrupted for 5 min at 50 Hz with a TissueLyzer II (QIAGEN) in order to better capture *Bifidobacteria*^51^, and after subsequent centrifugation, the procedure was automatized using the QIAamp PowerFecal DNA program for stool and biosolid on a QIAcube. DNA concentrations were measured with Qubit dsDNA HS Assay kits (Thermo Fisher Scientific, Waltham, MA) according to the manufacturer’s instructions, and DNA was stored at −20°C.

#### 16S rRNA gene sequencing

The 16S Metagenomic Sequencing Library Preparation, Preparing 16S Ribosomal RNA Gene Amplicons for the Illumina MiSeq System (Part # 15044223 Rev. B) was used to prepare the 16S RNA gene amplicons, which uses the primer pairs 341F/805R targeting the V3-V4 hypervariable region of 16S rRNA genes^39^ and the Nextera XT Index Kit (Illumina, San Diego, CA). The V3-V4 hypervariable region is widely applied in gut microbiota studies and considered among the least biased^40^, it is also recognized as a good region for a proper detection of *Bifidobacteria*^51^. The amplicon PCR protocol was modified to 30 cycles. The final pooled normalized libraries (4 nM), including a DNA mock control (ATCC MSA-2002), were diluted and denatured to 10 pM and spiked with 20% PhiX library (10 pM). A paired-end 300 bp sequencing run (600 cycles) was performed using the MiSeq platform (Illumina), with MiSeq Reagent Kit v3 chemicals.

Demultiplexed .fastq files were quality-filtered and trimmed, adaptor-trimmed and PhiX-filtered using bbduk.sh bbmap/38.08^41^. Reads were quality-trimmed to Q35 on the 5′ end and to Q30 on the 3′ end using Phred algorithm, sequences with lengths between 100 to 300 bp were retained. Phred scores were examined using MultiQC^42^. Trimmed sequences were further processed following the DADA2 Workflow with dada2 version 1.10.1 (https://benjjneb.github.io/dada2/tutorial.html ^43^) to generate an ASV table and assign taxonomy using a Naive Bayes classifier trained on the V3-V4 region of reference sequences (99% similarity) from SILVA version 132 (https://www.arb-silva.de). Merged reads with a length between 100 and 500 bp were kept and pseudo pooling was used for sample inference. ASVs identified as Archaea (2 ASVs), Eukaryote (119 ASVs) and Cyanobacteria (5 ASVs), as well as ASVs that were not identified at kingdom level (102 ASVs) were filtered out, as well as ASVs detected in only one sample and with less than 30 reads. After ASV filtering, we obtained a total of 33,883,590 sequencing reads, which belonged to 4,547 ASVs (Table S8). Two outliers (a 1w-sample in the *L. reuteri* group with 674,453 reads and a 3w-sample in the placebo group with 216,935 reads) were rarefied to the same number of reads as the sample with the third-most number of reads (134,932 reads).

#### Synthetic mock community

A synthetic mock microbial community (20 Strain Even Mix Whole Cell Material (ATCC® MSA-2002)) was prepared alongside the samples and it was used to determine the prevalence filtering threshold (amplicon sequences variants (ASV) detected in only one sample and with less than 30 reads) in order to remove potential contaminants (Table S7).

#### DNA extraction from L. reuteri cultures

To obtain *L. reuteri* DNA for standard curves in qPCR assays, *L. reuteri* DSM 17938 bacteria (courtesy of Dr Stefan Roos) were cultured anaerobically in 10 mL De Man, Rogosa, and Sharpe (MRS) broth at 36°C for 24 h, and DNA was extracted using the EZ1 DNA Tissue kit (QIAGEN). For DNA extraction, broth cultures were centrifuged at 3,000 rpm for 10 min at 4°C. Pellets were resuspended in 500 μL buffer G2, transferred to glass bead tubes, and shaken with a TissueLyzer II for 1 min at 30 Hz. Two hundred μL lysate were subjected to automatized DNA extraction using the protocol for purification of DNA from bacterial culture samples on an EZ1 Advanced XL robot (QIAGEN). DNA concentrations were measured with Qubit dsDNA HS Assay kits (Thermo Fisher Scientific, Waltham, MA) according to the manufacturer’s instructions, and DNA was stored at −20°C.

#### Quantitative PCR

The single-copy gene *Lactobacillus reuteri* unknown extracellular protein *lr1694* (GenBank accession number: DQ074924.1) is specific for the supplemented probiotic *L. reuteri* strain used in this study^52^. The *lr1694* gene was amplified in 20 μL qPCR reactions consisting of 2 μL of 10-fold diluted DNA, 1X SsoFast™ EvaGreen® Supermix (Bio-Rad, Hercules, CA), 300 μM of forward primer (sequence: 5′ TTAAGGATGCAAACCCGAAC 3′) and 300 μM reverse primer (sequence: 5′ CCTTGTCACCTGGAACCACT 3′). The qPCR assays were performed in CFX96™ Real-Time PCR Detection Systems (Bio-Rad) using the following program: 2 min at 98°C, 40 cycles of 5 s at 98°C and 5 s at 63°C. After each qPCR run, a melting curve analysis was conducted by gradually increasing the temperature from 65°C to 95°C in steps of 0.5°C. Serial dilutions of *L. reuteri* DSM 17938 DNA were used for generating standard curves with 5 × 10^4^ to 2.5 × 10^1^ *lr1694* gene copies/μL corresponding to quantification limits of 2.3 × 10^4^ to 4.5 × 10^7^ bacteria/g wet feces. Similar standard curve values were obtained in all runs (n = 24) (mean (95% confidence interval)): slope = −3.51 (−3.55 – −3.47), y-intercept = 42.3 (42.0 – 42.6), efficiency = 93% (91% – 95%), and R^2^ = 0.996 (0.995 – 0.997). Samples were analyzed in duplicates and they were re-run if the difference in Cq values from duplicates was larger than 0.3. Non-diluted or 20-fold diluted samples were used if Cq values fell outside of the range of the standard curve. If a non-diluted sample had a Cq value larger than 35, the sample was considered as negative for *L. reuteri* DSM 17938. Unspecific amplification or PCR inhibition were not observed. Data was normalized to the amount of feces used for DNA isolation and expressed as *L. reuteri* DSM 17938 bacteria per 1 g wet feces.

### Quantification and statistical analysis

#### Background and clinical characteristics

Continuous variables with skewed distributions were analyzed with Mann-Whitney U tests, while Student’s t tests were employed for continuous variables with normal distributions. Pearson’s chi-square test was used for categorical outcome variables. Fisher’s exact test was used when the observed frequency for any cell was less than five. Statistical analyses were performed in R Console 3.5.0.

#### 16S rRNA gene statistical analyses

Prior to β-diversity analyses, variance stabilizing transformation (VST) was applied for normalization across samples^46^, using the *DESeq2* package in R Console 3.5.0^44^. Bacterial community distributions across the *L. reuteri* and placebo groups were displayed by non-metric multidimensional scaling (NMDS) plots, and statistically tested using the analysis of similarities (ANOSIM), with 999 permutations^53^. Alpha-diversity was calculated using Shannon’s diversity index, Pielou’s evenness index, and richness assessed as number of observed ASVs, using the *diverse* package^45^, and statistically tested for differences between the groups (*L. reuteri versus* placebo, NEC cases versus matched controls, sepsis versus matched controls) using Mann-Whitney U tests. Rarefaction tests prior to α-diversity analysis were performed, concluding that rarefaction was not needed for the analysis (Table S9). Inference of differential abundance between the study groups was performed at ASV level as well as at all the different taxonomic levels, using the Linear discriminant analysis Effect Size (LEfSE)^54^. Correlation between α-diversity and growth parameters was explored using simple linear regression, and co-variation between β-diversity and growth parameters as well as qPCR data was assessed by fitting the growth clinical output data onto the ordination derived from the NMDS, with the envfit*(*) function. The microbial causal mediation effect on the growth parameters was tested using the Sparse Microbial Causal Mediation Model (SparseMCMM)^55^. The p values for the β-diversity analyses were corrected for false discovery rate according to Benjamini & Hochberg.

#### Quantitative PCR statistical analyses

Statistically significant differences in prevalence of *L. reuteri* DSM 17938 in the two study groups were tested for using Fisher’s exact test. Mann-Whitney U tests were performed to test for differences in *L. reuteri* DSM 17938 abundance in the two supplementation groups. A Kruskal-Wallis test with Dunn post hoc test was used to compare the abundance of *L. reuteri* DSM 17938 in *L. reuteri*-supplemented infants across time points. The p values for the qPCR analyses were corrected for false discovery rate according to Benjamini & Hochberg. Statistical analyses were performed in R Console 3.5.0.

### Additional resources

The study is registered at ClinicalTrials.gov (ID NCT01603368).

## Supplemental information

Document S1. Tables S1–S9 and Figure S1

Table S10. Metadata and qPCR data for all infant samples, related to STAR methods, subsection Data and code availability

Document S2. Article plus supplemental information
