# Supplementary material for: Effects of Lactobacillus reuteri supplementation on the gut microbiota in extremely preterm infants in a randomized placebo-controlled trial
Source: Cell Rep Med. 2021 Feb 22;2(3):100206. doi: 10.1016/j.xcrm.2021.100206 (PMC7974321; doi:10.1016/j.xcrm.2021.100206)
Supplement: Document S1. Tables S1–S9 and Figure S1 [file mmc1.pdf]

Cell Reports Medicine, Volume *2*

Supplemental information

Effects of *Lactobacillus reuteri* supplementation on the gut microbiota in extremely preterm infants in a randomized placebo-controlled trial

Magalí Martí, Johanne E. Spreckels, Purnika Damindi Ranasinghe, Erik Wejryd, Giovanna Marchini, Eva Sverremark-Ekström, Maria C. Jenmalm, and Thomas Abrahamsson

Cell Reports Medicine

Supplemental Information

Effects of *Lactobacillus reuteri* DSM 17938 supplementation on the gut microbiota in extremely preterm infants in a randomised placebo-controlled trial

Magalí Martí, Johanne E. Spreckels, Purnika Damindi Ranasinghe, Erik Wejryd, Giovanna Marchini, Eva Sverremark-Ekström, Maria C Jenmalm and Thomas Abrahamsson

This file includes:

Table S1: Background and clinical characteristics of the preterm infants. Related to Table 1.
Table S2: Alpha diversity in relation to Sepsis, NEC and antibiotic treatment at week 4. Related to Figure 2.
Table S3: Relative abundance of differentially abundant taxa between the two group. Related to Figure 4.
Table S4: Prevalence and mean relative abundance of the pathogens. Related to Figure 4.
Table S5: Estimated microbial mediation effect. Related to Figure 6.
Table S6: ASVs significantly contributing to the variance of the microbial community composition. Related to Figure 3.
Table S7: Genera detected in the mock community that are not part of the standard. Related to STAR Methods.
Table S8: Raw data preprocessing steps. Related to STAR Methods.
Table S9: Alpha-diversity analysis after three different rarefaction tests. Related to STAR Methods.
Figure S1: Taxonomic composition in relation to Sepsis and NEC. Related to Figure 4.

Table S1 Background and clinical characteristics of the extremely preterm infants from which samples were collected at week 1, week 2, week 3, and week 4 of life, and at post-menstrual week (PMW) 36+0 and at two years of age of age. Related to Table 1.

		**week 1**			**week 2**			**week 3**		
**Variables**	**Statistical test**	**Placebo n = 54**	***Lactobacillus reuteri* n = 54**	***P* value**	**Placebo n = 55**	***Lactobacillus reuteri* n = 54**	***P* value**	**Placebo n = 51**	***Lactobacillus reuteri* n = 51**	***P* value**
Gestational age, weeks, mean (SD)	Student t-test	25.5 (1.3)	25.5 (1.3)	0.89	25.5 (1.3)	25.6 (1.3)	0.842	25.5 (1.2)	25.6 (1.2)	0.717
Birth weight, g, median (IQR)	Mann-Whitney U test	763 (197.8)	727.5 (172.2)	0.150	765 (208.5)	711 (178.5)	0.366	761 (213.5)	729 (153)	0.451
Birth weight z-score, median (IQR)	Mann-Whitney U test	-0.8 (1.3)	-0.9 (1.7)	0.252	-0.8 (1.2)	-0.9 (1.6)	0.381	-0.9 (1.3)	-1 (1.5)	0.359
Birth length, cm, median (IQR)	Mann-Whitney U test	33.5 (3.5)	33 (3.8)	0.222	33 (3)	32.5 (3.8)	0.290	33 (4)	33 (3)	0.504
Birth length z-score, median (IQR)	Mann-Whitney U test	-0.8 (1.6)	-1.6 (2.3)	0.063	-0.9 (1.8)	-1.4 (2.3)	0.110	-0.9 (1.8)	-1.5 (2.2)	0.139
Birth head circumference, cm, median (IQR)	Mann-Whitney U test	23 (3)	23 (2)	0.117	23 (3)	23 (2)	0.391	23 (2.5)	23 (2)	0.277
Birth head circumference z-score, mean (SD)	Student t-test	-0.7 (0.8)	-1 (0.8)	0.060	-0.7 (0.8)	-0.9 (0.8)	0.165	-0.7 (0.8	-0.9 (0.8)	0.067
Apgar score at 5 minutes, median (IQR)	Mann-Whitney U test	7 (2)	7 (4)	0.683	6 (3)	7 (4)	0.558	7 (2.8)	7 (4)	0.832
Apgar score at 10 minutes, median (IQR)	Mann-Whitney U test	8 (2)	8 (2.8)	0.344	8 (2)	8 (2)	0.905	8 (2)	8 (2)	0.468
Small for gestational age (weight <2 SD), n (%)	Pearson's X2 test	10 (19%)	17 (31%)	0.182	10 (18%)	17 (31%)	0.166	10 (20%)	16 (31%)	0.256
Gender female/male, n (%)	Pearson's X2 test	19 (35%) / 35 (65%)	31 (57%) / 23 (43%)	**0.034**	20 (36%) / 35 (64%)	30 (56%) / 24 (44%)	0.069	18 (35%) / 33 (65%)	29 (57%) / 22 (43%)	**0.047**
Infants from multiple pregnancy, n (%)	Pearson's X2 test	17 (31%)	17 (31%)	1.000	18 (33%)	24 (44%)	0.289	17 (33%)	23 (45%)	0.311
Caesarean section, n (%)	Pearson's X2 test	30 (56%)	40 (74%)	0.070	31 (56%)	41 (76%)	0.051	30 (59%)	42 (82%)	**0.017**
Maternal smoking, n (%)	Fisher's exact test	4 (7%)	4 (7%)	1.000	4 (7%)	4 (7%)	1.000	4 (8%)	3 (6%)	1.000
Preeclampsia, n (%)	Fisher's exact test	4 (7%)	5 (9%)	1.000	4 (7%)	5 (9%)	0.742	5 (10%)	5 (10%)	1.000
Chorioamnionitis, n (%)	Pearson's X2 test	8 (15%)	14 (26%)	0.232	10 (18%)	16 (30%)	0.239	8 (16%)	16 (31%)	0.102
Preterm premature rupture of membranes, n (%)	Pearson's X2 test	13 (24%)	19 (35%)	0.292	14 (25%)	18 (33%)	0.488	13 (25%)	17 (33%)	0.514
Maternal antibiotics, n (%)	Pearson's X2 test	27 (50%)	33 (61%)	0.333	30 (55%)	32 (59%)	0.762	25 (49%)	32 (63%)	0.232
Antenatal corticosteroids, n (%)	Fisher's exact test	53 (98%)	53 (98%)	1.000	54 (98%)	53 (98%)	1.000	51 (100%)	50 (98%)	1.000
Inclusion site – Stockholm/Linköping, n (%)	Pearson's X2 test	35 (65%) / 19 (35%)	35 (65%) / 19 (35%)	1.000	34 (62%) / 21 (38%)	36 (67%) / 18 (33%)	0.743	31 (61%) / 20 (39%)	33 (65%) / 18 (35%)	0.838
Treatment with antibiotics within the actual week, n (%)	Pearson's X2 test	54 (100%)	54 (100%)	NA	43 (78%)	45 (83%)	0.661	34 (67%)	35 (69%)	1
Total days on antibiotics, median (IQR)	Mann-Whitney U test	7 (0)	7 (0)	0.927	11 (6)	12 (5.8)	0.534	14 (11.5)	17 (11)	0.595
Total days with insuline, median (IQR)	Mann-Whitney U test	0 (0)	0 (0)	0.519	0 (0)	0 (1.5)	0.981	0 (0)	0 (1)	0.672
Insulin within the actual week, n (%)	Fisher's exact test	9 (17%)	12 (22%)	0.628	13 (24%)	13 (24%)	1	6 (12%)	6 (12%)	1
Total days with corticosteroids, median (IQR)	Mann-Whitney U test	0 (0)	0 (0)	0.407	0 (0)	0 (0)	0.263	0 (0)	0 (0)	0.351
Corticosteroids within the actual week, n (%)	Fisher's exact test	4 (7%)	2 (4%)	0.679	5 (9%)	2 (4%)	0.438	9 (18%)	8 (16%)	1

Variables	Statistical test	week 4 Placebo n = 48	week 4 *Lactobacillus reuteri* n = 53	week 4 *P* value	PMW36 Placebo n = 41	PMW36 *Lactobacillus reuteri* n = 50	PMW36 *P* value	2 years Placebo n = 27	2 years *Lactobacillus reuteri* n = 20	2 years *P* value
Gestational age, weeks, mean (SD)	Student t-test	25.5 (1.3)	25.5 (1.1)	0.866	25.6 (1.1)	25.5 (1.2)	0.807	25.3 (1.3)	25.4 (1.2)	0.799
Birth weight, g, median (IQR)	Mann-Whitney U test	760.5 (212.8)	730 (162)	0.561	761 (177)	727.5 (182.2)	0.418	720 (245.5)	730 (155.2)	0.830
Birth weight z-score, median (IQR)	Mann-Whitney U test	-0.9 (1.4)	-0.9 (1.8)	0.566	-0.6 (2)	-0.9 (1.2)	0.407	-0.9 (1.1)	-1 (1.5)	0.991
Birth length, cm, median (IQR)	Mann-Whitney U test	33 (3)	33 (3)	0.681	33 (3)	32 (3.8)	0.202	33 (4)	33 (4)	0.688
Birth length z-score, median (IQR)	Mann-Whitney U test	-0.9 (2)	-1.4 (2.3)	0.397	-0.7 (1.6)	-1.5 (2.1)	0.117	-1.1 (1.5)	-1 (1.8)	1.000
Birth head circumference, cm, median (IQR)	Mann-Whitney U test	23 (3)	23 (2)	0.253	23 (1)	23 (2)	0.167	23 (3.5)	23 (2)	0.752
Birth head circumference z-score, mean (SD)	Student t-test	-0.7 (0.9)	-1 (0.9)	0.117	-0.7 (0.9)	-0.9 (0.8)	0.152	-0.6 (0.8)	-0.8 (0.8)	0.349
Apgar score at 5 minutes, median (IQR)	Mann-Whitney U test	7 (3)	7 (4)	0.953	6.5 (3)	7 (5)	0.762	NR	NR	NR
Apgar score at 10 minutes, median (IQR)	Mann-Whitney U test	8 (2)	8 (2)	0.869	8 (3)	8 (2)	0.434	NR	NR	NR
Small for gestational age (weight <2 SD), n (%)	Pearson's X2 test	10 (21%)	18 (34%)	0.212	11 (27%)	14 (28%)	1.000	NR	NR	NR
Gender female/male, n (%)	Pearson's X2 test	17 (35%) / 31 (65%)	27 (51%) / 26 (49%)	0.170	14 (34%) / 27 (66%)	27 (54%) / 23 (46%)	0.093	10 (37%) / 17 (63%)	10 (50%) / 10 (50%)	0.555
Infants from multiple pregnancy, n (%)	Pearson's X2 test	15 (31%)	21 (40%)	0.503	7 (17%)	22 (44%)	**0.012**	NR	NR	NR
Caesarean section, n (%)	Pearson's X2 test	29 (60%)	41 (77%)	0.104	24 (59%)	39 (78%)	0.076	NR	NR	NR
Maternal smoking, n (%)	Fisher's exact test	5 (10%)	4 (8%)	0.733	1 (2%)	4 (8%)	0.374	NR	NR	NR
Preeclampsia, n (%)	Fisher's exact test	6 (12%)	5 (9%)	0.753	4 (10%)	6 (12%)	1.000	NR	NR	NR
Chorioamnionitis, n (%)	Pearson's X2 test	7 (15%)	15 (28%)	0.154	6 (15%)	18 (36%)	**0.039**	NR	NR	NR
Preterm premature rupture of membranes, n (%)	Pearson's X2 test	11 (23%)	19 (36%)	0.229	9 (22%)	21 (42%)	0.072	NR	NR	NR
Maternal antibiotics, n (%)	Pearson's X2 test	25 (52%)	34 (64%)	0.305	19 (46%)	32 (64%)	0.140	NR	NR	NR
Antenatal corticosteroids, n (%)	Fisher's exact test	47 (98%)	53 (100%)	0.475	40 (98%)	50 (100%)	0.451	NR	NR	NR
Inclusion site – Stockholm/Linköping, n (%)	Pearson's X2 test	29 (60%) / 19 (40%)	35 (66%) / 18 (34%)	0.705	27 (66%) / 14 (34%)	37 (74%) / 13 (26%)	0.538	NR	NR	NR
Treatment with antibiotics within the actual week, n (%)	Pearson's X2 test	24 (50%)	38 (72%)	**0.042**	NA	NA	NA	NA	NA	NA
Total days on antibiotics, median (IQR)	Mann-Whitney U test	17 (18)	22 (17)	0.565	21 (20)	26 (23)	0.037	NA	NA	NA
Total days with insuline, median (IQR)	Mann-Whitney U test	0 (1.25)	0 (2)	0.878	NA	NA	NA	NA	NA	NA
Insulin within the actual week, n (%)	Fisher's exact test	3 (6%)	4 (8%)	1	NA	NA	NA	NA	NA	NA
Total days with corticosteroids, median (IQR)	Mann-Whitney U test	0 (3.25)	0 (0)	0.233	NA	NA	NA	NA	NA	NA
Corticosteroids within the actual week, n (%)	Fisher's exact test	0 (100%)	0 (100%)	NA	NA	NA	NA	NA	NA	NA

Apgar score is missing from one infant in the *L. reuteri* group. NA: not applicable. NR: not relevant. IQR: inter-quartile range.

Table S2 Bacterial diversity (Shannon index), richness (observed AVS), and evenness (Pielou's evenness index), for the antibiotic treatment *vs* no antibiotic treatment at week 4, as well as for the necrotizing enterocolitis (NEC) and sepsis cases in comparison with the matched controls. Related to Figure 2.

	Diversity		Richness		Evenness	
**Antibiotic treatment at week 4**	**AB+**	**AB-**	**AB+**	**AB-**	**AB+**	**AB-**
mean	1.3	1.6	22.6	26.7	0.4	0.5
median	1.4	1.6 **	23.0	27 *	0.5	0.5
SEM	0.1	0.1	0.9	1.3	0.0	0.0
IQR	0.8	0.5	7.8	13.5	0.2	0.2
N	62	39	62	39	62	39
**Treated with antibiotic at week 4**	**L. reuteri**	**Placebo**	**L. reuteri**	**Placebo**	**L. reuteri**	**Placebo**
mean	1.6	1.0	23.8	20.6	0.5	0.3
median	1.6 ***	1.0	24.0	21.0	0.5 ***	0.3
SEM	0.1	0.1	1.2	1.4	0.0	0.0
IQR	0.5	0.6	6.8	10.2	0.2	0.2
N	38	24	38	24	38	24
**Not treated with antibiotic at week 4**	**L. reuteri**	**Placebo**	**L. reuteri**	**Placebo**	**L. reuteri**	**Placebo**
mean	1.7	1.6	28.5	25.5	0.5	0.5
median	1.8	1.6	28.0	25.0	0.5	0.5
SEM	0.2	0.1	2.0	1.7	0.0	0.0
IQR	0.7	0.6	11.0	12.5	0.2	0.1
N	15	24	15	24	15	24
**NEC**	**NEC**	**control**	**NEC**	**control**	**NEC**	**control**
mean	1.7	1.4	25.7	20.4	0.6	0.5
median	1.9	1.6	30.0	21.0	0.5	0.5
SEM	0.2	0.1	5.4	2.4	0.1	0.1
IQR	0.4	0.4	21.0	12.3	0.1	0.3
N	7	14	7	14	7	14
**Culture proven sepsis**	**Sepsis**	**control**	**Sepsis**	**control**	**Sepsis**	**control**
mean	1.5	1.2	23.2	22.4	0.5	0.4
median	1.5	1.3	22.5	24.0	0.5	0.4
SEM	0.1	0.1	1.9	1.0	0.0	0.0
IQR	0.9	0.5	12.0	10.3	0.2	0.1
N	28	56	28	56	28	56

Table S3 Mean relative abundance percentage of the taxa (Phyla, Family and Genera) that significantly differed in relative abundance between the two groups. Related to Figure 4.

Taxa	Timepoint	Placebo	*L. reuteri*
Proteobacteria	1w	21.30	5.46
Staphylococcaceae	1w	62.29	35.11
Lactobacillaceae	1w	0.09	45.46
Lactobacillaceae	2w	0.09	45.46
Lactobacillaceae	3w	1.14	18.25
Lactobacillaceae	4w	0.94	14.95
Lactobacillaceae	5w	0.39	0.84
Staphylococcus	1w	62.29	35.11
Lactobacillus	1w	0.09	45.45
Lactobacillus	2w	0.66	24.51
Lactobacillus	3w	1.14	18.25
Lactobacillus	4w	0.94	14.95
Lactobacillus	5w	0.37	0.84

Table S4 Prevalence and mean relative abundance of the pathogens found in the data set. Related to Figure 4.

	week 1				week 2				week 3			
	Prevalence (N)		Relative abundance (mean)		Prevalence (N)		Relative abundance (mean)		Prevalence (N)		Relative abundance (mean)	
**Pathogen**	Placebo (n = 54)	*L. reuteri* (n= 54)	Placebo (n = 54)	*L. reuteri* (n= 54)	Placebo (n = 55)	*L. reuteri* (n= 54)	Placebo (n = 55)	*L. reuteri* (n= 54)	Placebo (n = 51)	*L. reuteri* (n= 51)	Placebo (n = 51)	*L. reuteri* (n= 51)
Acinetobacter	0	1	0.0	0.0	4	8	1.0	2.3	6	5	0.3	2.2
Campylobacter	0	0	0.0	0.0	0	2	0.0	0.0	0	1	0.0	0.5
Citrobacter	0	0	0.0	0.0	1	0	0.4	0.0	2	0	0.2	0.0
Enterobacter	8	6	0.9	0.2	17	18	2.1	1.2	26	29	2.6	2.4
Enterococcus	22	19	10.5	6.0	43	37	15.4	7.9	44	44	17.7	14.0
Escherichia/Shigella	18	14	7.2	2.8	17	10	5.2	3.5	30	30	9.7	9.7
Haemophilus	1	0	0.0	0.0	6	6	0.1	0.3	18	18	0.7	1.0
Klebsiella	8	6	1.8	0.7	21	17	7.6	1.8	15	23	8.8	4.8
Proteus	0	0	0.0	0.0	1	0	0.3	0.0	2	4	0.0	1.6
Pseudomonas	2	1	1.6	0.0	4	1	1.0	0.0	1	6	0.0	0.1
Salmonella	0	0	0.0	0.0	0	0	0.0	0.0	0	0	0.0	0.0
Serratia	0	0	0.0	0.0	0	1	0.0	0.6	1	2	0.0	0.0
Staphylococcus	54	50	62.3	35.1	55	54	48.9	38.8	51	50	34.9	17.8
Streptococcus	8	4	0.9	0.2	18	19	2.6	0.8	14	12	0.8	0.2
Ureaplasma	3	7	0.4	2.2	2	1	0.0	0.0	0	1	0.0	0.0

	week 4				PMW36				2 years			
	Prevalence (N)		Relative abundance (mean)		Prevalence (N)		Relative abundance (mean)		Prevalence (N)		Relative abundance (mean)	
**Pathogen**	Placebo (n = 48)	*L. reuteri* (n= 53)	Placebo (n = 48)	*L. reuteri* (n= 53)	Placebo (n = 41)	*L. reuteri* (n= 50)	Placebo (n = 41)	*L. reuteri* (n= 50)	Placebo (n = 27)	*L. reuteri* (n= 20)	Placebo (n = 27)	*L. reuteri* (n= 20)
Acinetobacter	6	7	1.6	1.0	1	4	0.1	0.0	0	0	0.0	0.0
Campylobacter	0	0	0.0	0.0	1	0	0.0	0.0	2	0	0.0	0.0
Citrobacter	2	0	0.0	0.0	7	4	1.2	0.4	1	0	0.0	0.0
Enterobacter	24	19	6.2	1.9	28	25	4.2	2.9	2	1	0.0	0.0
Enterococcus	42	47	16.6	8.0	39	45	6.6	4.9	4	1	0.0	0.0
Escherichia/Shigella	31	33	13.0	10.8	30	39	13.6	20.5	13	9	1.3	1.4
Haemophilus	15	14	0.6	1.1	15	30	0.2	1.8	7	3	0.0	0.0
Klebsiella	23	14	9.9	4.4	29	35	14.1	8.2	1	2	0.1	0.0
Proteus	4	3	0.5	1.2	1	1	0.0	0.0	0	0	0.0	0.0
Pseudomonas	3	3	2.2	0.0	3	0	0.0	0.0	0	0	0.0	0.0
Salmonella	0	1	0.0	0.0	0	0	0.0	0.0	0	0	0.0	0.0
Serratia	1	1	0.1	0.0	2	1	1.0	0.0	0	0	0.0	0.0
Staphylococcus	45	52	22.8	23.6	32	40	0.6	0.9	1	1	0.0	0.0
Streptococcus	20	13	0.7	0.4	21	24	0.1	0.3	9	12	0.3	0.4
Ureaplasma	0	0	0.0	0.0	0	0	0.0	0.0	0	0	0.0	0.0

Table S5 Estimated microbial mediation effect. Related to Figure 6.

Time point	Growth parameter	Estimated *P*-values		Estimated casual effects		
		OME	CME	ME	DE	TE
week 1	Weight day 14	0.818	0.727	0.140	-0.298	-0.158
	Weight day 28	1.000	1.000	0.661	-0.575	0.087
	Weight PMW36	0.333	0.667	0.757	-1.014	-0.258
	Head day 14	0.545	0.545	0.758	-0.313	0.445
	Head day 28	0.727	0.727	0.803	-0.366	0.437
	Head PMW36	0.667	0.667	0.681	-0.296	0.385
	Length day 14	0.909	0.455	-0.009	0.028	0.019
	Length day 28	1.000	0.727	0.068	0.153	0.221
	Length PMW36	1.000	1.000	0.499	-0.605	-0.105
week 2	Weight day 28	0.636	0.909	0.091	0.046	0.137
	Weight PMW36	0.727	0.727	0.410	-0.731	-0.320
	Head day 28	0.727	0.727	0.402	0.009	0.411
	Head PMW36	0.727	0.909	0.102	-0.259	-0.157
	Length day 28	0.818	0.909	-0.096	0.509	0.412
	Length PMW36	0.909	1.000	0.151	-0.027	0.124
week 3	Weight day 28	0.727	0.727	-0.419	1.070	0.651
	Weight PMW36	0.273	0.273	-1.022	1.690	0.668
	Head day 28	0.727	0.818	0.257	0.332	0.589
	Head PMW36	0.545	0.545	0.868	-0.365	0.503
	Length day 28	0.727	0.818	0.872	-0.635	0.237
	Length PMW36	0.455	0.455	0.767	-0.402	0.365
week 4	Weight PMW36	0.273	0.364	-0.687	0.588	-0.099
	Head PMW36	0.909	0.909	0.275	-0.051	0.224
	Length PMW36	0.636	0.909	0.278	-0.293	-0.015

OME: tests the overall mediation effect of the microbiome community.
CME: test whether at least one taxon has a mediation effect.
ME: estimates of the overall microbial mediation effect
DE: estimates of the direct treatment effect.
TE: estimates of the total treatment effect.

Table S6 ASVs that significantly contributed to the variance explained (*envfit();* $p < 0.01$ and $R^2 > 0.3$) of the bacterial community composition. Related to Figure 3.

**Timepoint**	**ASV**	**NMDS1**	**NMDS2**	**pVal**	**r2**	**Taxa**
1w	ASV_60	-21.98296	-23.791359	0.001	0.4197117	Lactobacillus
1w	**ASV_115**	**-21.61612**	**-24.301643**	**0.001**	**0.4231307**	**Lactobacillus**
1w	ASV_142	-21.39567	-23.953471	0.001	0.4126174	Lactobacillus
1w	ASV_54	-21.3842	-23.916403	0.001	0.4117113	Lactobacillus
1w	ASV_2306	-21.25109	-22.478338	0.001	0.3827538	Lactobacillus
1w	ASV_99	-20.8442	-23.530428	0.001	0.3952646	Lactobacillus
1w	ASV_1773	-20.75729	-21.768915	0.001	0.3619003	Lactobacillus
1w	ASV_428	-20.08627	-21.575789	0.001	0.3475891	Lactobacillus
1w	ASV_1787	-20.05051	-20.472619	0.001	0.3284605	Lactobacillus
1w	ASV_429	23.20261	18.459696	0.001	0.3516486	Staphylococcus
1w	ASV_556	24.68473	16.580184	0.001	0.3536953	Staphylococcus
1w	ASV_9	25.16905	10.800848	0.001	0.3000558	Staphylococcus
1w	ASV_480	26.60004	9.741569	0.001	0.3209841	Staphylococcus
1w	ASV_1255	26.74682	-15.038307	0.001	0.3766172	Lactobacillus
1w	ASV_1228	27.2955	-7.510071	0.001	0.3205782	Lactobacillus
1w	ASV_1325	27.82421	-13.839526	0.001	0.3862877	Lactobacillus
1w	ASV_7	28.20744	22.504851	0.001	0.5208512	Staphylococcus
1w	**ASV_1**	**30.52605**	**26.783029**	**0.001**	**0.6596682**	**Staphylococcus**
1w	ASV_17	37.22087	-16.447531	0.001	0.6623657	Lactobacillus
1w	ASV_5	38.5338	-14.406019	0.001	0.6769548	Lactobacillus
1w	ASV_16	38.75567	-13.440752	0.001	0.6730624	Lactobacillus
1w	ASV_13	39.13333	-14.71464	0.001	0.6991751	Lactobacillus
1w	**ASV_8**	**39.86317**	**-15.380725**	**0.001**	**0.7302558**	**Lactobacillus**
2w	ASV_5	31.635318	2.979022	0.001	0.4038672	Lactobacillus
2w	ASV_8	32.113762	0.8443705	0.001	0.4128027	Lactobacillus
2w	ASV_13	32.133603	5.3434177	0.001	0.4244482	Lactobacillus
2w	ASV_16	33.306896	6.0986387	0.001	0.4586171	Lactobacillus
2w	**ASV_17**	**34.561489**	**4.1525932**	**0.001**	**0.4846962**	**Lactobacillus**
2w	**ASV_469**	**-27.836324**	**5.3831789**	**0.001**	**0.3215358**	**Klebsiella**
3w	**ASV_27**	**-29.224532**	**17.4241**	**0.001**	**0.463069**	**Staphylococcus**
3w	ASV_104	-23.330591	14.68713	0.001	0.3040114	Lactobacillus
3w	**ASV_15**	**-5.694603**	**-27.66607**	**0.001**	**0.319136**	**Klebsiella**
3w	ASV_4	23.350047	-22.33923	0.001	0.4177064	Staphylococcus
3w	ASV_1	27.23989	-17.57323	0.001	0.420332	Staphylococcus
3w	ASV_17	32.575855	22.79617	0.001	0.6323407	Lactobacillus
3w	ASV_8	33.507301	22.16962	0.001	0.6456924	Lactobacillus
3w	ASV_16	34.902925	22.13418	0.001	0.6832545	Lactobacillus
3w	**ASV_5**	**35.027534**	**22.93229**	**0.001**	**0.7011273**	**Lactobacillus**
3w	ASV_13	35.302795	19.6912	0.001	0.6536123	Lactobacillus
4w	**ASV_13**	**-40.435394**	**2.729943**	**0.001**	**0.6569895**	**Lactobacillus**
4w	ASV_8	-39.780061	1.599692	0.001	0.6340049	Lactobacillus
4w	ASV_17	-38.996714	2.770995	0.001	0.6113688	Lactobacillus
4w	ASV_5	-38.538443	2.327821	0.001	0.5962521	Lactobacillus
4w	ASV_16	-37.287542	6.182177	0.001	0.571432	Lactobacillus
4w	ASV_616	-30.724109	2.640385	0.001	0.380377	Lactobacillus
4w	ASV_646	-29.637233	5.069486	0.001	0.3616261	Lactobacillus
4w	**ASV_384**	**-17.34843**	**-23.301276**	**0.001**	**0.337567**	**Staphylococcus**
4w	**ASV_6**	**-1.469882**	**27.943947**	**0.001**	**0.3132099**	**Escherichia/Shig**

Table S7 Genera detected in the mock community that are not part of the standard, thus considered potential contaminants. This was the basis to determine the threshold for the prevalence filtering, which was a compromise between the amount of potential contaminants detected and the amount of true taxa removed from the analysis. Related to STAR Methods.

Genus	Prevalence	Number of reads	mock1	mock2	mock3	mock4	mock5	mock6	mock7	mock8	mock9
Allorhizobium-Neorhizobium-Pararhizobium-Rhizobium	1	2	0	0	0	2	0	0	0	0	0
Azorhizophilus	1	1	0	1	0	0	0	0	0	0	0
Enterobacter	1	17	0	0	0	0	0	17	0	0	0
Hungatella	1	2	0	0	0	0	0	0	2	0	0
Klebsiella	2	19	0	0	0	10	0	0	9	0	0
Veillonella *	3	83	0	10	0	0	38	35	0	0	0

* Veillonella is not removed in the filtering step by prevalence.

Table S8 Raw data preprocessing steps. Related to STAR Methods.

	raw ASV table			Filtered taxa[1]		Filtered prevalence[2]		Rarefaction[3]		Rarefaction[3]			
	n samples	n taxa	n reads	n taxa	n reads	n taxa	n reads	n taxa	n reads	n reads		average reads	
**All**	558	4775	33899242	4547	33883590	3322	33871208	3322	33249777	*L. reuteri*	Placebo	*L. reuteri*	Placebo
**1w**	108	690	5739773	633	5736500	492	5735248	491	5195761	2240974	2954787	41499.5	54718.3
**2w**	109	822	8431686	716	8427377	566	8425997	566	8425997	4263467	4162530	78953.1	75682.4
**3w**	102	962	5653934	938	5650195	753	5648684	753	5566740	2606540	2960200	51108.6	58043.1
**4w**	101	1017	7031415	987	7029335	798	7027672	798	7027672	3722744	3304928	70240.5	68852.7
**PMW36**	91	981	4814752	965	4814079	778	4812098	778	4812098	2760846	2051252	55216.9	50030.5
**2y**	47	1761	2227682	1744	2226104	1326	2221509	1326	2221509	950179	1271330	47508.9	47086.3
**All L.reuteri**	282	2848	16557280	2725	16550812	2112	16544750	2112	16544750	16544750	NA	58669.3	NA
**All Placebo**	276	2966	17341962	2854	17332778	2202	17326458	2202	16705027	NA	16705027	NA	60525.5

PMW36: post-menstrual week 36+0.
Filtered taxa: amplicon sequence variants (ASVs) identified as Archaea (2 ASVs), Eukaryote (119 ASVs) and Cyanobacteria (5 ASV), as well as ASVs that were not identified at Kingdom (102 ASV).
Filtered prevalence: ASVs detected in only one sample and with less than 30 reads were filtered out.
Rarefaction: two samples had much more reads than the rest (1 week sample in the *L. reuteri* group with 674453 reads and a 3 week sample in the placebo group with 216935 reads). These were rarefied to the same number of reads as the next one (134932 reads).

Table S9 Alpha-diversity analysis after three different rarefaction tests: No rarefaction, rarefaction to the minimum sequencing depth and to 10% of the maximum sequencing depth. Based on these results, the no rarefaction options was chosen for the data analysis in this paper. The statistical test applied was Mann-Whitney U test and p-values were adjusted using the Benjamini & Hochberg method. Related to STAR Methods.

Timepoint	Sample read counts	p-adjusted (FDR)			Samples removed from analysis
		Diversity	Richness	Evenness	
1w	No rarefaction	0.00	0.00	0.00	0
	380 (min seq depth)	0.00	0.00	0.00	0
	13493 (10% of max depth)	0.00	0.01	0.00	11 *L. reuteri* + 10 placebo
2w	No rarefaction	0.00	0.00	0.00	0
	1690 (min seq depth)	0.00	0.00	0.00	0
	13492 (10% of max depth)	0.00	0.00	0.00	3 placebo
3w	No rarefaction	0.00	0.00	0.03	0
	1582 (min seq depth)	0.00	0.00	0.10	0
	13493 (10% of max depth)	0.00	0.00	0.06	3 *L. reuteri* + 2 placebo
4w	No rarefaction	0.00	0.23	0.00	0
	427 (min seq depth)	0.00	0.01	0.00	0
	12561 (10% of max depth)	0.00	0.09	0.01	3 *L. reuteri* + 1 placebo
5w	No rarefaction	0.68	0.41	0.25	0
	1171 (min seq depth)	0.59	0.60	0.24	0
	12742 (10% of max depth)	0.51	0.57	0.21	1 *L. reuteri* + 2 placebo
6w	No rarefaction	0.68	0.68	0.81	0
	296 (min seq depth)	0.59	0.60	0.77	0
	12014 (10% of max depth)	1.00	0.91	0.68	3 *L. reuteri* + 3 placebo

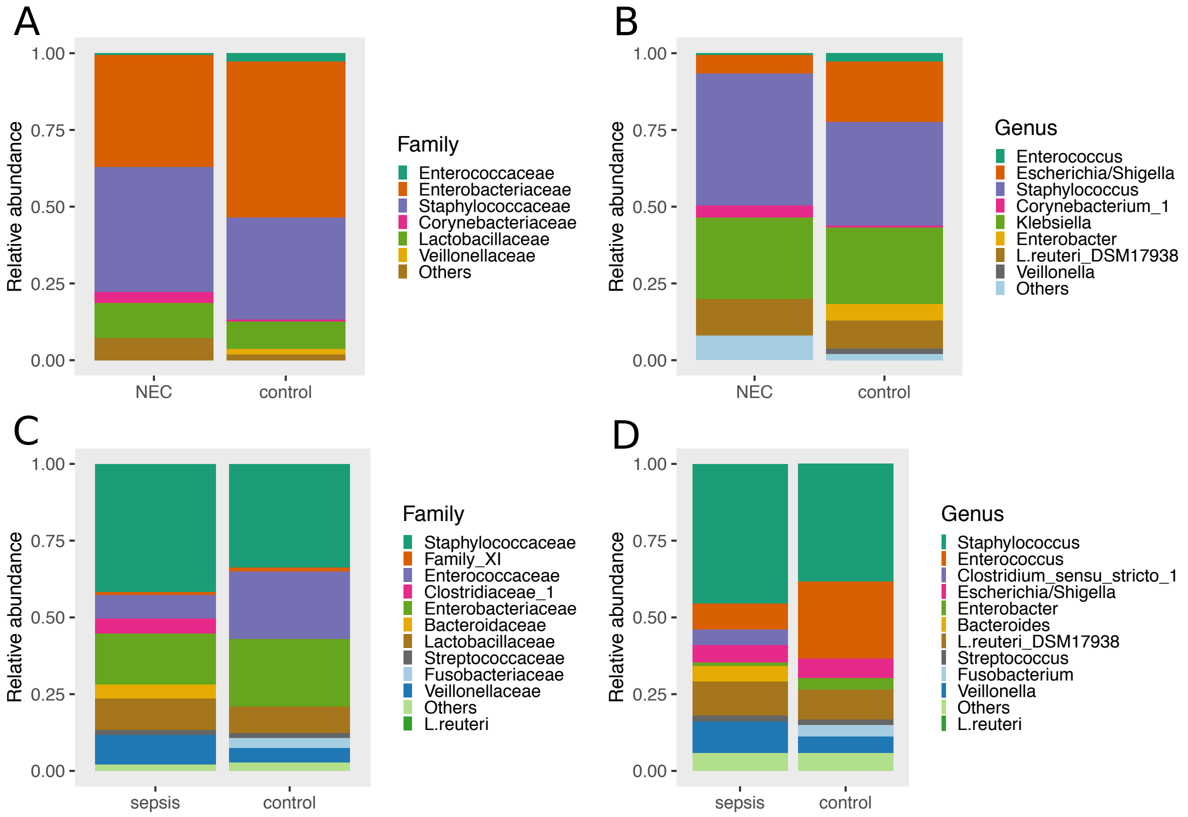

Figure S1 Taxonomic composition of the bacterial community in extremely low birth weight preterm infants comparing NEC cases with their matched controls at family (A) and genus (B) level, and sepsis cases with their matched controls at family (C) and genus level (D), respectively. Taxa with a relative abundance of < 1% across all samples are grouped in “Others”. Related to Figure 4.
